# Assessment of Radio-Induced Damage in Endothelial Cells Irradiated with 40 kVp, 220 kVp, and 4 MV X-rays by Means of Micro and Nanodosimetric Calculations

**DOI:** 10.3390/ijms20246204

**Published:** 2019-12-09

**Authors:** Nicolas Tang, Marta Bueno, Sylvain Meylan, Yann Perrot, Hoang N. Tran, Amélie Freneau, Morgane Dos Santos, Aurélie Vaurijoux, Gaëtan Gruel, Mario A. Bernal, Marie-Claude Bordage, Dimitris Emfietzoglou, Ziad Francis, Susanna Guatelli, Vladimir Ivanchenko, Mathieu Karamitros, Ioanna Kyriakou, Wook-Geun Shin, Sébastien Incerti, Carmen Villagrasa

**Affiliations:** 1 IRSN, Institut de Radioprotection et de Sûreté Nucléaire, BP17, 92262 Fontenay aux Roses, France; 2 SymAlgo Technologies, 75 rue Léon Frot, 75011 Paris, France; 3 Instituto de Fisica Gleb Wataghin, Universidade Estadual de Campinas, Campinas 13083-859, SP, Brazil; 4 INSERM, Université Paul Sabatier, UMR 1037, CRCT, 31330 Toulouse, France; 5 Université Toulouse III-Paul Sabatier, UMR 1037, CRCT, 31330 Toulouse, France; 6 Medical Physics Laboratory, University of Ioannina, Medical School, GR-45110 Ioannina, Greece; 7 Department of Physics, Faculty of Sciences, Université Saint Joseph, 1104 2020 Beirut, Lebanon; 8 Centre for Medical Radiation Physics, University of Wollongong, 2522 Wollongong, Australia; 9 Geant4 Associates International Ltd., Hebden Bridge HX7 7BT, UK; 10 LEPHE, Tomsk State University, 634050 Tomsk, Russia; 11 Université de Bordeaux, CNRS/IN2P3, Centre Nucléaires de Bordeaux Gradignan, CENBG, chemin du solarium, BP120, 33175 Gradignan, France; 12 CNRS/IN2P3, Centre d’Etudes Nucléaires de Bordeaux Gradignan, CENBG, chemin du solarium, BP120, 33175 Gradignan, France

**Keywords:** Monte Carlo simulation, Geant4/Geant4-DNA, X-rays, microdosimetry, nanodosimetry, DSB yield

## Abstract

The objective of this work was to study the differences in terms of early biological effects that might exist between different X-rays energies by using a mechanistic approach. To this end, radiobiological experiments exposing cell monolayers to three X-ray energies were performed in order to assess the yields of early DNA damage, in particular of double-strand breaks (DSBs). The simulation of these irradiations was set in order to understand the differences in the obtained experimental results. Hence, simulated results in terms of microdosimetric spectra and early DSB induction were analyzed and compared to the experimental data. Human umbilical vein endothelial cells (HUVECs) were irradiated with 40, 220 kVp, and 4 MV X-rays. The Geant4 Monte Carlo simulation toolkit and its extension Geant4-DNA were used for the simulations. Microdosimetric calculations aiming to determine possible differences in the variability of the energy absorbed by the irradiated cell population for those photon spectra were performed on 10,000 endothelial cell nuclei representing a cell monolayer. Nanodosimetric simulations were also carried out using a computation chain that allowed the simulation of physical, physico-chemical, and chemical stages on a single realistic endothelial cell nucleus model including both heterochromatin and euchromatin. DNA damage was scored in terms of yields of prompt DSBs per Gray (Gy) and per giga (10^9^) base pair (Gbp) and DSB complexity was derived in order to be compared to experimental data expressed as numbers of histone variant H2AX (γ-H2AX) foci per cell. The calculated microdosimetric spread in the irradiated cell population was similar when comparing between 40 and 220 kVp X-rays and higher when comparing with 4 MV X-rays. Simulated yields of induced DSB/Gy/Gbp were found to be equivalent to those for 40 and 220 kVp but larger than those for 4 MV, resulting in a relative biological effectiveness (RBE) of 1.3. Additionally, DSB complexity was similar between the considered photon spectra. Simulated results were in good agreement with experimental data obtained by IRSN (Institut de radioprotection et de sûreté nucléaire) radiobiologists. Despite differences in photon energy, few differences were observed when comparing between 40 and 220 kVp X-rays in microdosimetric and nanodosimetric calculations. Nevertheless, variations were observed when comparing between 40/220 kVp and 4 MV X-rays. Thanks to the simulation results, these variations were able to be explained by the differences in the production of secondary electrons with energies below 10 keV.

## 1. Introduction

For the evaluation of DNA damage induced by photon irradiation, most radiobiological experiments rely on the mean absorbed dose [1]. Hence, these experiments assume that a homogeneous dose equal to the macroscopic absorbed dose D is delivered to the cell population. However, due to the stochastic nature of energy depositions and the small volume of a given cell nucleus, the energy received from one cell to another may be significantly different in the irradiated population.

Moreover, although X-rays are considered low-linear energy transfer (LET) radiation [2], a higher relative biological effectiveness (RBE) at very low-energy (≤30 keV) than at high energy has been reported in the literature. Indeed, very low energy X-rays may lead to higher risk estimates at low doses for many biological endpoints such as double-strand breaks (DSBs), chromosome aberrations, micronucleus formation, and cell survival [3,4,5,6,7,8,9,10,11,12,13,14,15,16,17,18,19,20,21,22]. This could be explained by the fact that energy depositions vary between these different photon energies since the type of interaction (photoelectric effect, Compton effect, or pair production), which causes the energy deposition depends on the photon energy [23]. In addition, the lower the photon energy, the lower the energy of the secondary electrons, meaning the stopping power of the latter is higher. The stochastic nature of the radiation–tissue interaction leads to differences in terms of energy received by each cell nucleus in a cell population [1], which can be analyzed through microdosimetry.

Differences in track structure and patterns of energy depositions can also impact DNA DSB induction and location [24,25], which itself can lead to differences in final cell fate. Indeed, it is well known that DSBs are among the most deleterious forms of DNA lesions and can lead to potential chromosome aberrations and cell death if unrepaired or misrepaired [26]. Yields of DSBs, as well as their spatial distribution and especially their proximity, play an important role in the formation of chromosome aberrations and other cellular endpoints such as cell death [24,25,27,28]. However, it should be noted that for the same biological endpoint of interest and radiation quality, RBE values from the literature can present great variability [29].

In this work, simulations were performed to reproduce experimental irradiations using different photon sources carried out at the IRSN (Institut de Radioprotection et de Sûreté Nucléaire), Fontenay aux Roses, France [30]. In Freneau et al. [30], human umbilical vein endothelial cells (HUVECs) were irradiated with 40 kVp, 220 kVp, and 4 MV X-rays. The experiments showed that the yield of histone variant H2AX (γ-H2AX) foci was similar for 40 kVp and 220 kVp but higher when compared to 4 MV for a dose of 2 Gray (Gy). It has been pointed out that the secondary electron energy spectrum is more likely to explain differences in the yields of γ-H2AX foci rather than the initial photon energy spectrum. Measurements of γ-H2AX foci can be related to the yield of DSB, although certain precautions must be taken. Indeed, the usual ratio of 1 DSB:1 γ-H2AX focus [31] is not entirely accurate when high-LET radiation is considered and given the size of the γ-H2AX foci, which is about 0.2 μm^²^ as reported by Rothkam and Horn [32]. In this case, several DSBs can be contained within a single γ-H2AX focus [33]. However, this ratio remains more or less accurate for low-LET radiation such as photon irradiation, as DSBs are less close [34].

The calculation of energy depositions by Monte Carlo simulation can then be useful to understanding the observations made in radiobiological experiments since DNA damage and energy depositions are closely linked.

The simulations in this work were performed by means of a computation chain that has been extensively described in [35] and which is based on the Geant4 [36,37,38] Monte Carlo simulation toolkit (version 10.1) and its Geant4-DNA extension [39,40,41,42].

As a first approach, microdosimetric quantities were calculated, such as the frequency distribution of the specific energy and the microdosimetric spread, which reflects the spread in specific energy per cell in an irradiated cell population, since these quantities could be of use to study the possible difference between radiation qualities in terms of RBE [43].

In order to evaluate the influence of radiation quality on DSB induction, nanodosimetric calculations were performed using the computation chain in order to assess the yields of DSB per unit absorbed dose and per Gbp, taking into account direct and indirect damage as well as DSB complexity [35]. Then, the number of simulated DSBs was correlated with measurements of γ-H2AX foci. For these calculations, a model representing an endothelial cell nucleus filled with around 6 Gbp [44] of DNA composed of heterochromatin and euchromatin in the Gap0/Gap1 (G0/G1) phase of the cell cycle was generated with DnaFabric [45,46] software and used in the simulation.

## 2. Results

### 2.1. Secondary Electron Spectra

**Figure 1:**
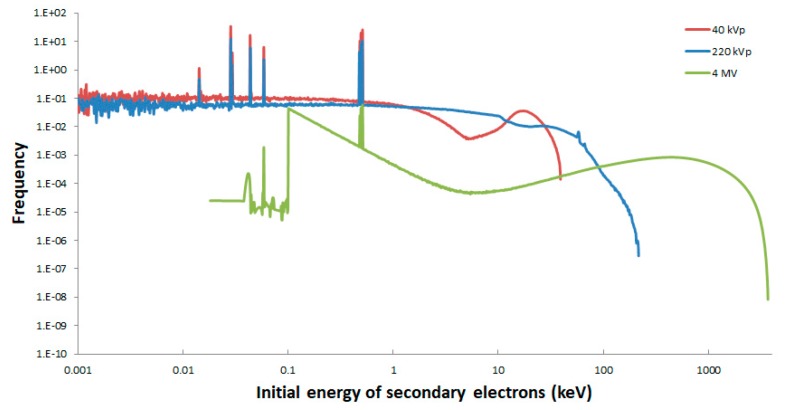
Frequency of secondary electrons per unit energy in log scale resulting from 40 kVp (red line), 220 kVp (blue line), and 4 MV (green line) X-rays.

Proportions in photoelectric effect (PE), Compton effect (CE), and pair production (CONV) were scored and compared between the three photon energy spectra. For 40 kVp X-rays, the photoelectric effect was found to be dominant (68.9%), the whereas Compton effect represented 31.1% of the photon interactions, as presented in Table 1. As the photon energy increases, the photoelectric effect decreases and the Compton effect increases, becoming dominant (the two latter effects represented 19.6% and 80.4%, respectively, for 220 kVp X-rays). At higher energies, i.e., 4 MV here, the conversion process can be observed, although at a very low rate (PE = 3.3%, CE = 96.3%, and CONV = 0.4%). In principle, it could be assumed that these variations could lead to differences in terms of energy depositions because this could give rise to different secondary electron spectra. Nevertheless, it can be seen in Figure 1 that the secondary electron spectra calculated at the cell positions did not appear as expected from our first hypothesis. Indeed, the photon spectra are different, but we can observe that the distributions of secondary electrons resulting from both 40 and 220 kVp X-rays are quite similar, as well as their mean energies (9.8 and 18.4 keV, respectively), if we account for the fact that the acceleration potential increases almost six times. Conversely, the secondary electron spectrum for the 4 MV X-rays shows relatively large differences, especially in its mean energy, which is 858.4 keV. As a consequence, from these three secondary electron spectra, we were able to expect variations in energy depositions, in particular between 40 or 220 kVp and 4 MV. Furthermore, a sudden decrease can be seen in Figure 1 in the frequency of secondary electrons at 100 eV for 4 MV. This is not a physical phenomenon but a bias resulting from the simulation and is due to the default production threshold of 100 eV of Livermore. Indeed, when an electron reached 100 eV in the simulation, its energy was deposited locally and its transport was stopped. As a result, electrons with an energy of less than 100 eV that had not reached yet the cell layer or had not been created directly inside it were not recorded. However, this decrease was not observed for 40 and 220 kVp, considering that the majority of electrons (85% and 68%, respectively) were created directly inside the cell layer volume. By contrast, in the case of the 4 MV X-ray configuration, 98% of the electrons were created at the bottom surface of the cell layer. As a result, the vast majority of electrons with an energy of less than 100 eV were not recorded in the phase space file, except for those created directly inside the cell layer. Nevertheless, this cut-off energy of 100 eV did not have any impact on the calculation of the energy deposition in the cell nuclei. Indeed, cell nuclei of 2 μm in thickness were placed in the middle of the layer which was 5 μm in height (in z). The distance separating them from each surface was then 1.5 μm. As this distance was larger than the range of 100 eV electrons, which was found to be 4 nm ± 2 nm in our calculation, these electrons could not reach the cell nuclei.

### 2.2. Microdosimetric Calculations

**Figure 2:**
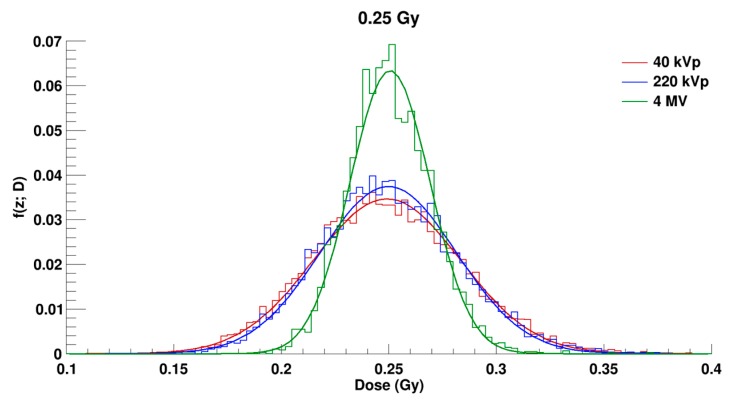
f (z; D) distributions in the irradiated cell population for a dose of 0.25 Gray (Gy) for 40 kVp (in red), 220 kVp (in blue), and 4 MV (in green) X-rays. Bars correspond to the histograms obtained in the simulations and the solid curves correspond to the distributions fitted with a Gaussian function.

Distributions of the specific energy f (z; D) in the irradiated cell population for a dose of 0.25 Gy for 40, 220 kVp, and 4 MV are shown in Figure 2. In this figure it is possible to observe that the f (z; D) distributions correspond to normal distributions centered at the simulated dose of 0.25 Gy. The distributions are very much alike between 40 and 220 kVp, while the distribution is narrower in the case of the 4 MV X-rays.

**Figure 3:**
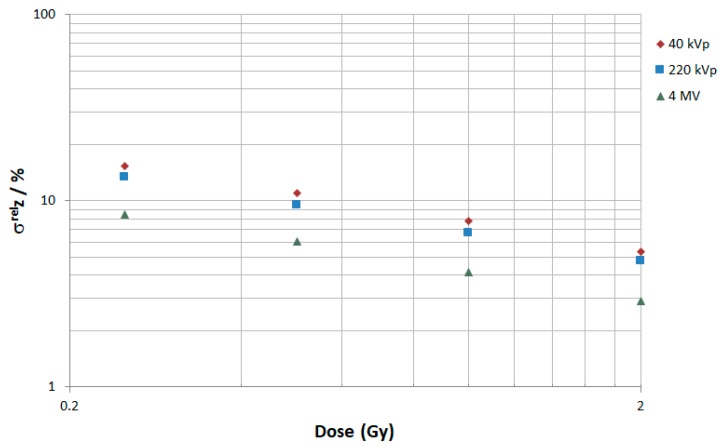
Microdosimetric spread $\sigma_{z}^{\mathrm{rel}}$ (in %) for 40 kVp (red diamonds), 220 kVp (blue squares), and 4 MV (green triangles) at 0.25, 0.5, 1, and 2 Gy.

Figure 3 shows the microdosimetric spread ($\sigma_{z}^{\mathrm{rel}}$) calculated with Equation (4) (see Section 4.3.1) in the irradiated cell population for the three photon energy spectra as a function of the simulated doses 0.25 Gy, 0.5 Gy, 1 Gy, and 2 Gy, as used in the experiments [30]. As can be observed, the $\sigma_{z}^{\mathrm{rel}}$ decreases when the dose increases. This was expected, since the total number of individual energy depositions increases in each of the cell nucleus volume.

### 2.3. Yields of DSB/Gy/Gbp and DSB Complexity

The yields of DSB/Gy/Gbp computed by the number of DSB divided by the dose received and by the 6 Gbp contained in the cell nucleus are presented in Table 2 for chemistry stage simulation end-times of 2.5 ns and 10 ns. Errors correspond to the standard deviation (SD) of the mean over 10 batches.

### 2.4. Comparison between Simulated Results and Experimental Data

**Figure 4:**
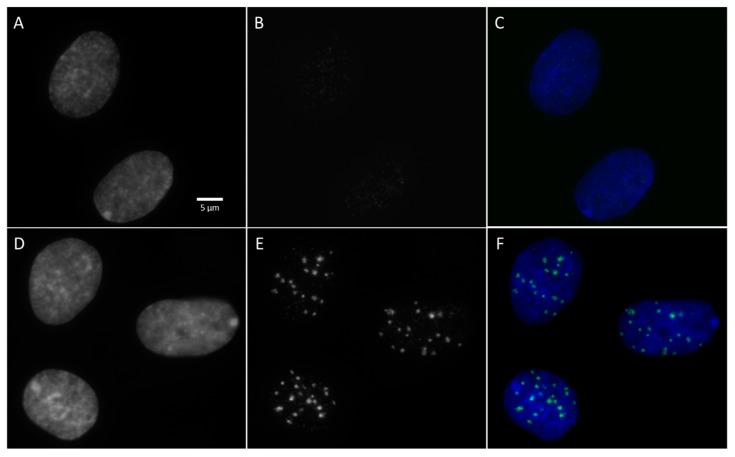
Labeling by immunofluorescence of the phosphorylation of the serine 139 of histone variant H2AX (γ-H2AX) for non-irradiated cells (**A**,**B**,**C**), and 30 min after irradiation of human umbilical vein endothelial cells (HUVECs) with 1 Gy of 4 MV X-rays delivered by medical linear accelerator (Elekta Synergy^®^) (**D**,**E**,**F**). (**A**) and (**D**) correspond to nuclear DNA labelled with 4′,6-diamidino-2-phenylindole (DAPI). (**B**) and (**E**) correspond to the immunostaining of the phosphorylation of serine 139 of γ-H2AX. (**C**) and (**F**) correspond to the merging of the images obtained for DNA and γ-H2AX labelling.

The experimental number of γ-H2AX foci per nucleus, determined by image analysis as shown in Figure 4**,** is compared to the simulated number of DSBs for chemistry stage simulation end-times of 2.5 and 10 ns in Table 3 for a dose of 1 Gy. The mean number of γ-H2AX foci per endothelial cell nucleus was observed experimentally 30 min post-irradiation for 0.25, 0.5, 1, and 2 Gy doses and obtained from at least three replicate experiments [30]. Errors correspond to the standard error. Endothelial cells were all synchronized in the G0/G1 phase of the cell cycle in order to minimize the dispersion in the cell DNA content during irradiation and to keep it close to the 6 Gbp used in the simulated cell nucleus phantom.

## 3. Discussion

The variations that can be seen in the secondary electron spectra between 40 kVp, 220 kVp, and 4 MV X-rays seem to explain the origin of the difference in RBE for DSB induction. The fact that electron spectra are very much alike between 40 and 220 kVp but different when compared to 4 MV could indicate a similar RBE value between 40 and 220 kVp but a higher RBE for the latter with respect to 4 MV. RBE for DSB induction increases drastically for electron energies around 1 keV and below for a maximum value of around 5, while it remains constant and close to 1 for electron energies above 10 keV [20]. Considering this, it is more convenient to examine the proportion of electrons below 10 keV than the ones above 10 keV. In Figure 1, we can clearly observe that the proportion of low-energy electrons (≤ 10 keV) is almost the same between 40 and 220 kVp and much lower at 4 MV, indicating a possible difference in RBE.

From the microdosimetric point of view, $\sigma_{z}^{\mathrm{rel}}$, which reflects the spread of the specific energy received per nucleus in a cell population, was relatively similar when comparing between 40 and 220 kVp. Indeed, for a macroscopic dose of 0.25, 0.5, 1, or 2 Gy, the difference when comparing between 40 and 220 kVp was always below 2%. However, this difference was higher when comparing between 40/220 kVp and 4 MV, e.g., 7% at 0.25 Gy. This can be explained by the fact that the mean energy deposited by an electron track in a cell nucleus is lower for 4 MV X-rays than for 40 and 220 kVp X-rays (1.8 mGy, 1.6 mGy, and 0.4 mGy for 40 kVp, 220 kVp, and 4 MV, respectively) since electrons from 4 MV X-rays are more energetic, and, thus, their mean free path is larger. As a result, more tracks are required for 4 MV X-rays to obtain the same macroscopic dose, which leads to a reduction in statistical dispersion.

DSB induction was equivalent between 40 and 220 kVp. Indeed, as can be seen in Table 2, the simulated DSB yield was 3.5 ± 0.3 DSB/Gy/Gbp for both photon spectra. However, the DSB yield decreased to 2.8 ± 0.3 DSB/Gy/Gbp for 4 MV X-rays. In a review of experimental DSB yield carried out by Prise et al. [18], data obtained for mammalian cells using pulsed-field gel electrophoresis (PFGE) techniques ranged from 5.8 to 6.0 DSB/Gy/Gbp for 225 and 250 kV X-rays, and from 4.2 to 6.9 DSB/Gy/Gbp for ^60^Co, which is consistent with our results, especially since for the γ-H2AX technique, the repair processes had already been initiated. However, the simulation of DSB yield has led to higher values in other studies. Indeed, in Friedland et al. [4], the authors’ calculation was found to be 8.8 ± 1.4 DSB/Gy/Gbp using 220 kVp X-rays.

In our simulation, an RBE of 1.3 for DSB induction was observed for 40 or 220 kVp with respect to 4 MV, which is similar to simulated RBEs reported earlier [29].

It should be noted that the scoring method for DNA damage can strongly influence the final yield of simulated DSB induction. As mentioned in our previous works [35,46,47], a change in the parameters to score direct and/or indirect strand breaks can greatly increase the yield of DSB. Moreover, a chemical simulation end-time of 10 ns has been suggested in our computation chain instead of the 2.5 ns currently used when considering the cell nucleus model filled with heterochromatin and euchromatin [46]. Hence, simulations were also performed with a parameter of 10 ns and the number of DSBs showed an increase by a factor of ~1.3 with respect to those obtained with 2.5 ns (Table 2). Although the chemical simulation end-time was changed, these DSB yields (3.6 to 4.7 DSB/Gy/Gbp for 10 ns) are still in agreement with published data. Furthermore, the RBE value did not change, which indicates that the number of DSBs increased in the same manner for the three radiation qualities.

Another important point to underline here is that, as mentioned in the Materials and Methods section, in these simulations only the strand breaks of the DNA were explicitly scored. Nevertheless, as reported in [44,48,49,50,51], complex DNA damage that is at the origin of high mutagenic or carcinogenic potential also includes non-DSB oxidative clustered DNA lesions (OCDL). Moreover, as reported in a previous work [48], post-irradiation repair of some sugar and base residues can produce additional strand breaks that can convert some of these non-DSB clustered lesions into DSBs, changing the total number of detected DSBs after irradiation compared to those predicted by the simulation.

From the experiments led at IRSN [30], we observed in Table 3 that the mean number of γ-H2AX foci per nucleus 30 min post irradiation increased with the dose for each photon spectrum. No significant difference was observed in that study between 40 and 220 kVp. However, significant differences were observed between 40 or 220 kVp relative to 4 MV for a dose of 2 Gy, which is in line with the results showed in the simulation. It is also important to note that the number of DSBs per nucleus at 1 Gy obtained using the simulation was always higher than the number of γ-H2AX foci obtained experimentally (Table 3). In addition, as mentioned above, γ-H2AX foci were observed 30 min post irradiation. Between the time of irradiation and observation of the γ-H2AX foci, DSB repair has already been initiated and some foci have already disappeared [31]. The kinetics of foci formation and disappearance may also differ depending on the level of compaction of the chromatin. Falk et al. [31] have even suggested that DSBs occurring in euchromatin could be repaired without causing the formation of γ-H2AX foci, and, even if this assumption is quite questionable, rapid repair of DSBs in euchromatin regions tends to decrease the number of foci detected with respect to the number of DSBs that can be obtained by simulation. Besides, from our simulation results, it can be seen that about 55% of the DSBs occurred in the euchromatin regions and 45% in the heterochromatin ones for the three X-rays energies considered in the present work. It should also be noted that overlapping phenomena can occur between different foci during microscopy detection [52]. In the end, it appears that the number of foci is experimentally underestimated [53]. We can see in Table 3 that the number of DSBs simulated per nucleus at 2.5 ns seemed to be close to the number of γ-H2AX foci. The increase in the number of simulated DSBs for a chemical simulation end-time of 10 ns showed that this time would be better adapted in the simulation for the calculation of DSBs. Despite difficulties in the direct comparison of the absolute number of simulated DSBs and experimental γ-H2AX foci, we can observe that the relative comparison of the number of DSBs between the three X-rays energies is in good agreement with that of γ-H2AX foci. Our RBE value of 1.3 between 40/220 kVp and 4 MV is similar to that in other works [12,54] where the RBE values for DSB induction were 1.15 and 1.1 for 29 kVp X-rays and 125 kVp X-rays with respect to ^60^Co. Nevertheless, an RBE value for DSB induction of 1.1 with respect to ^60^Co was reported in this study, which is in line with our 1.3 RBE value when comparing 220 kVp with 4 MV X-rays. In general, RBE values between 40–250 kVp X-rays and higher energies, e.g., ^60^Co, have been found to be in the range ~1.12–1.53 [55,56,57,58].

Concerning DSB complexity as defined in the Materials and Methods section, i.e., only taking into account the number of strand breaks included in the cluster, DSB complexity was found to be similar between the considered X-ray energies. Indeed, as can be seen in Table 2, the proportions of simple and complex DSB were about 86% and 14% for all the radiation qualities. In Liang et al. [21] it was reported that DSB complexity increases with decreasing photon energy with a maximum of around 1 keV. Nevertheless, it was also mentioned that DSB complexity was similar for soft and conventional X-rays as well as for ^60^Co γ-rays when base damage was not considered. However, DSB complexity presented a larger difference when base damage simulated in the chemical stage was taken into account. In that sense, as has been pointed out above, an important improvement in the computation chain will consist of being able to better simulate and consider base damage as well as different oxygen concentrations in the cell nucleus medium. These improvements are being currently developed by the Geant4-DNA collaboration and will be available in future public releases. These improvements are needed in order to simulate not only DSBs but also non-DSB clustered damage in more realistic cellular media and will allow for extending simulations to repair processes in a correct manner. With the same objective, the use of a new standard to record DNA damage (standard DNA damage) [59] which has been recently proposed can also simplify inter-code comparisons of DNA damage induction and facilitate simulation extension to chromosome aberration computations or other late effects.

## 4. Materials and Methods

### 4.1. Simulation of Experimental Cell Irradiations

#### 4.1.1. Experimental Data on γ-H2AX Foci

Primary HUVECs from the Lonza Group (ref. C2519A, lot0000394986) and isolated by Lonza from human tissue (from two females and two males) were donated after permission and obtained for their use in research applications by informed consent or legal authorization. HUVECs were handled as described in [30]. Briefly, G0/G1-phase synchronized HUVECs were obtained by contact inhibition induced in confluent culture. Synchronized cells were seeded 5 h prior to irradiation at a density of 30,000 cells/cm² on plastic dishes (1-well Permanox^®^ in Nunc^®^ Lab-Tek^®^ chamber slide systems; Thermo Fisher Scientific, Waltham, Massachusetts, USA) and incubated at 37 °C. Then, cells were irradiated using two irradiation facilities at IRSN, Fontenay aux Roses, France; these were a small animal radiation research platform (SARRP, XSTRAHL Ltd., Camberley, England) and a medical linear accelerator (Elekta Synergy^®^, Stockholm, Sweden). With the SARRP, the irradiations were performed with 40 kVp X-rays and 220 kVp X-rays while the medical linear accelerator was used to deliver 4 MV X-rays, as detailed elsewhere [30]. Both simulated setups are described hereafter. After exposure to X-rays, HUVECs were immunostained for the in situ detection of phosphorylation of the serine 139 of γ-H2AX, and DNA was stained with 4′,6-diamidino-2-phenylindole (DAPI). A detailed process for this has been previously described in [30]. In addition to the synchronization of cells in the G0/G1 phase prior to radiation exposure, the average number of γ-H2AX foci per endothelial cell nucleus was determined specifically in the nucleus in G0/G1 using the methodology described in [1]. Briefly, thousands of images were acquired on an inverted Olympus IX81 fluorescence microscope with a UPLSAPO 100XO oil immersion objective (Olympus, Tokyo, Japan). For each channel, images were acquired as 3 z-stack layers around and including the focus plane with a step size of 0.5 µm between planes. The images of the 3D stack were projected to 2D xy images using maximum intensity projection. Image analysis was then performed with ScanR analysis software (Olympus, version 2.8.1). An edge segmentation algorithm was used to detect nuclei and γ-H2AX foci. A first selection of relevant nuclei was based on their area and circularity. This step allowed us to consider only isolated nuclei by removing from the analysis objects corresponding to nucleus clusters. To isolate nuclei in the G0/G1 phase, a second level of selection was based on the DNA content of each nucleus (related to the integrated intensity levels of DAPI fluorescence) and the whole nucleus level of γ-H2AX (associated with the S-phase of the cell cycle). The average number of γ-H2AX foci per endothelial cell nucleus was measured for doses of 0.25, 0.5, 1, and 2 Gy 30 min after irradiation. These doses were selected to remain in the range of values where the fluorescence technique showed linearity with the dose and where the intrinsic noise of foci in G0/G1 cells remained low compared to the radio-induced signal. On the other hand, only experimental data at 30 min post irradiation were compared here to simulated data. Indeed, it is assumed that the number of detectable γ-H2AX foci reach a maximum at 30 min after irradiation [1]. Therefore, and as the simulation tool did not take into consideration any repair process, we focused on the post-irradiation time which maximizes the number of detectable DNA damage on the basis of a γ-H2AX foci methodology. The number of γ-H2AX foci was obtained from at least three identical experiments (replicates) for each configuration with approximately 4000 cells analyzed each time [30].

#### 4.1.2. SARRP Configuration

**Figure 5:**
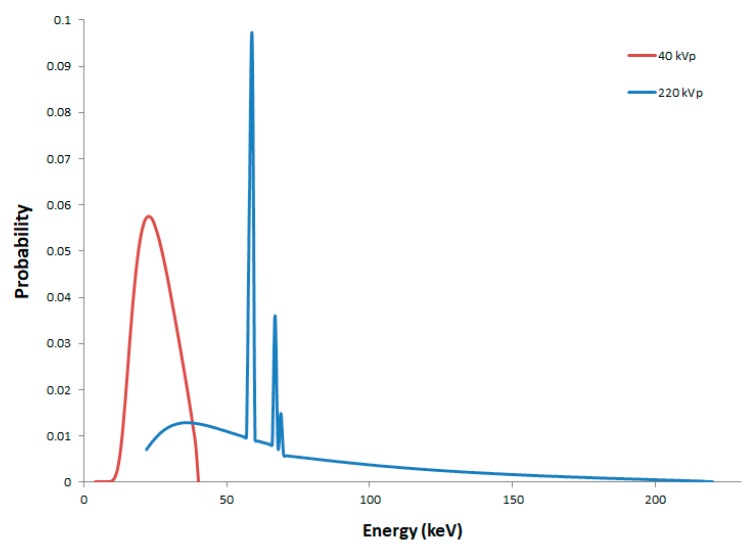
Photon energy spectra after inherent and additional filtrations for 40 kVp (in red) and 220 kVp (in blue) X-rays corresponding to small animal radiation research platform (SARRP) irradiations. Spectra were obtained with SpekCalc software [60,61,62].

As mentioned in our previous work [30], the SARRP configuration makes use of an inherent filtration of 0.8 mm of beryllium and an additional filtration of 1 mm of aluminum, which results in the suppression of low-energy photons. The initial photon energy spectra after inherent and additional filtrations corresponding to 40 and 220 kVp X-rays used in this work are shown in Figure 5. The spectra were calculated using SpekCalc software [60,61,62]. The half-value layer (HVL) values of aluminum were 0.852 mm and 5.420 mm [63] and the resulting mean photon energies were 25.6 keV and 70.2 keV for 40 kVp and 220 kVp, respectively. The dose rate used was about 1 Gy.min^−1^ expressed in terms of Kerma in air at 30.5 cm from the source.

**Figure 6:**
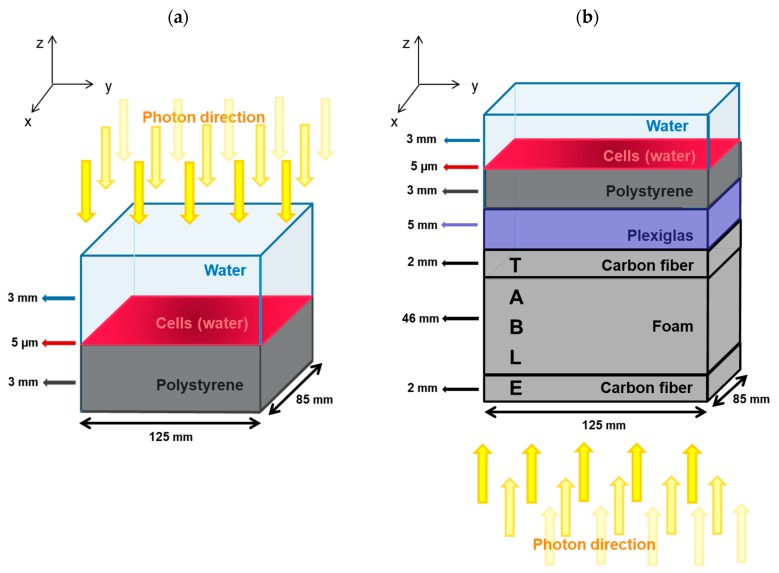
(**a**) Setup used for simulation for SARRP irradiation. The cell culture chamber was modeled by three layers of material (from top to bottom): 3 mm of liquid water representing the cell culture medium, 5 µm of liquid water defining the cell layer, and 3 mm of polystyrene modeling the chamber material. The dimensions in the x–y plane of each layer were 125 mm × 85 mm. Photons were generated in a parallel beam emitted from above and directed towards the cell monolayer. (**b**) Setup for simulation of medical linear accelerator irradiation. The cell chamber (same as the one used in the SARRP simulation) was placed on a table made of carbon fiber and foam (the carbon fiber was 4 mm high and the foam was 46 mm high). Between the cell chamber and the table, a layer of 5 mm in height of Plexiglas^®^ was placed to ensure an electronic equilibrium. Photons were generated in a parallel beam from below the table towards the cell chamber.

The cell culture chamber (1-well Permanox^®^ in Nunc^®^ Lab-Tek^®^ chamber slide systems; Thermo Fisher Scientific, Waltham, Massachusetts, USA) was modeled by a polystyrene volume (1.06 g.cm^−3^) with a 3 mm height and a liquid water volume of 5 µm in height representing the cell layer, followed by a liquid water volume of 3 mm in height representing the cell culture medium, as depicted in Figure 6a. The dimensions in the plane of the cell culture (x–y plane) of each layer were 125 mm × 85 mm. In the SARRP configuration, photons were generated in a parallel beam emitted from above and directed perpendicular to the cell monolayer.

#### 4.1.3. Medical Linear Accelerator Configuration

**Figure 7:**
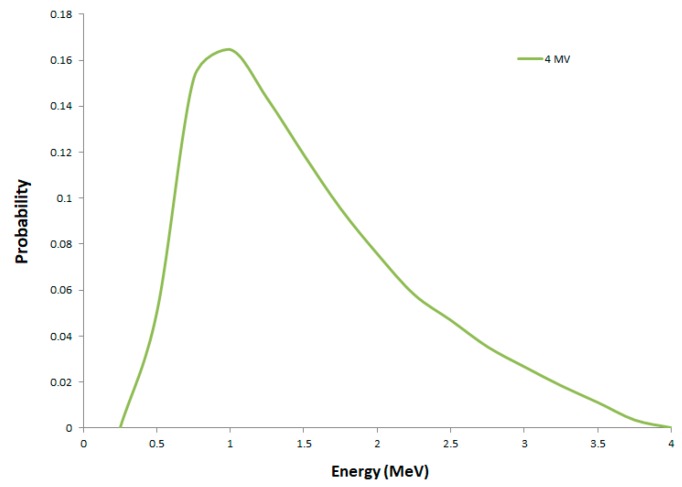
Photon energy spectrum for 4 MV X-rays [63].

For the medical linear accelerator simulation, a chamber containing cells (same as the one used in the SARRP configuration) was placed on a table made of a layer of carbon fiber (0.55 g.cm^−3^, 2 mm in height), then a layer of plastic foam (0.03 g.cm^−3^, 46 mm in height), and another layer of carbon fiber for the irradiation (2 mm in height). An additional layer of Plexiglas^®^ (1.19 g.cm^−3^, 5 mm in height) was placed between the cell chamber and the table to ensure electronic equilibrium in the cell layer. The dimensions in the x–y plane of each layer were set to 125 mm × 85 mm. In this configuration, photons were generated in a parallel beam from below the table towards the cell chamber, as represented in Figure 6b. The dose rate measured with an ionizing chamber calibrated in free-air Kerma was about 1 Gy.min^−1^ at a distance of 120 cm from the source and had a 30 cm × 30 cm irradiation field [30]. Concerning the source description, the 4 MV X-ray energy spectrum after the collimator used in the simulation is represented in Figure 7 and was taken from Sheikh-Bagheri et al. [64], leading to a mean photon energy of 1.3 MeV and a beam quality of 0.626 in terms of percentage depth dose at 10 cm in liquid water.

### 4.2. Phase Space of Secondary Electrons

The simulations in this work were constructed in two steps. The first one consisted of simulating the photon source at the macroscopic level as represented in Figure 6. The aim of this stage was to score a phase space in the 5 mm high cell layer, recording the position, momentum, and energy of every secondary electron reaching the cell layer. For this purpose, the Livermore low energy physics library of Geant4 was used to simulate photons and electrons. Electron transport was simulated down to a minimum energy of 100 eV by default. In addition, the Auger process was activated as its effect could have an impact on DSB damage, although this was not major [19]. This first stage is a crucial step in simulating radiation-induced DNA damage by photons, given the fact that the secondary electron spectrum will determine the energy depositions in the cell layer or cell nuclei [54].

The second part of the simulation corresponded to micro- or nanodosimetric simulation in the irradiated cells. The electrons that were scored during the first part were used as a particle source.

### 4.3. Microdosimetric and Nanodosimetric Approaches

#### 4.3.1. Microdosimetric Calculations in the Cell Population

The stochastic nature of energy depositions at the cell level leads to a spread of the dose per cell around the macroscopic irradiation dose D. The beam quality can have an effect on the proportion of cells that do not receive exactly the macroscopic dose, which can lead to possible differences in cell fate such as senescence or cell death.

This is why biological effects should be linked to the energy actually deposited in each cell nucleus rather than the macroscopic dose. Analysis of the energy deposition at the corresponding spatial scale can be achieved using microdosimetry, which gives the formalism [65,66] to describe the distribution of energy depositions that occur stochastically. Indeed, energy depositions in cell nuclei result from the combination of the number of tracks traversing the volume of interest as well as the energy deposited per track, with both having a statistical dispersion. From this stochastic property, different values of deposited energy are obtained in each cell nucleus, leading to different specific energies, z. The absorbed dose D corresponds to the ratio between the mean value of the deposited energy in the matter and the mass of the volume where the depositions occur.

Unlike D, z is a stochastic quantity with a probability density f(z). As a matter of fact, D represents the expected value of the specific energy, i.e.,

(1) $$D=\bar{z}=\int \mathrm{zf}\left(z;D\right)\mathrm{dz}$$

The probability density function used to obtain the specific energy z for a given macroscopic dose D in the volume of interest is represented by f(z; D), i.e.,

(2) $$f\left(z;D\right)=\sum_{\nu =0}^{\infty }p\left(\nu \right).f_{\nu }\left(z\right)$$

where

(3) $$p\left(\nu \right)=e^{-n}\frac{n^{\nu }}{v!}$$

The number of tracks ν leading to energy depositions in the target volume follows a Poisson distribution with mean value n, with n being the number of tracks going through the target volume.

It is therefore appropriate to consider the width of the distribution f(z) because it represents a variability of the energy absorbed by the irradiated cell population.

The relative standard deviation is equal to

(4) $$\sigma_{z}^{\mathrm{rel}}=\frac{\sigma_{z}}{D}$$

where σ_z_ is the standard deviation of the distribution f(z).

In this work, microdosimetric simulations were computed using the Geant4 Monte Carlo toolkit version 10.2 as well as its extension, Geant4-DNA. In particular, the G4EmDNAPhysics_option2 physics constructor from the Geant4-DNA extension was used which enables electron transport from 1 MeV down to 7.4 eV.

The cell layer dimensions were reduced and modeled by a 4 mm × 4 mm × 5 µm volume (x, y, and z respectively). The x–y coordinates of the electrons from the first part of the simulation that were outside 4 mm × 4 mm were uniformly sampled in order to be always contained in the cell layer volume. In this volume, 10,000 phantoms of endothelial cell nuclei were placed. Each phantom was modeled as an elliptical cylinder (semi-major axis = 9.5 µm, semi-minor axis = 5.1 µm, and height = 2.0 µm) with a total volume of 304.4 µm^3^ made of liquid water (without DNA constituents). These cell nucleus dimensions corresponded to the mean values of the irradiated cells as determined from the biological experiments. Individual energy depositions in each cell nucleus were scored and cumulated to derive the distribution f (z; D). The mean and the standard deviation $\sigma_{z}$ of the specific energy were then calculated. To compute the $\sigma_{z}^{\mathrm{rel}}$ value (in %), the standard deviation $\sigma_{z}$ was divided by the mean specific energy, which corresponded to the macroscopic dose D (Equation (4)).

To do this, during the simulation of the electron transport in the cell layer modeled by 10,000 endothelial cell nuclei, several quantities were recorded: the “event identifier”, which corresponded to a track generated by an electron, the nucleus identifier in the cell population, and the energy deposition for each electron inelastic interaction. From this data, it was then possible to calculate f (z; D) and the statistical spread of the specific energy z. To compute f (z; D), all energy depositions in each of the cell nuclei were cumulated. The initial macroscopic dose was 0.25 Gy. Then, the specific energy was cumulated by pairs of different cell nuclei relatively far enough to ensure superimposing independent tracks in order to get the f (z; D) distribution and the $\sigma_{z}^{\mathrm{rel}}$ value for higher doses (0.5 Gy, 1 Gy, and 2 Gy) without having to run additional and time-consuming simulations. However, this method resulted in decreasing the number of cell nuclei available in the cell population at each cumulating step and therefore could not be applied for higher doses.

#### 4.3.2. Nanodosimetric Simulations for the Calculation of Double-Strand Breaks

In this work, nanodosimetric calculations were also performed to compute DNA damage yields in order to study the possible difference in RBE in terms of DSB induction and characteristics between the three photon energy spectra. Then, the simulated results were compared to the experimental data obtained at IRSN, Fontenay aux Roses, France.

##### Simulation Configuration

In this part of the study we focused on a single endothelial cell nucleus model in the G0/G1 phase of the cell cycle containing the geometry representing ~6 Gbp that had been generated with DnaFabric software [45]. This cell nucleus model was filled with DNA with the proportions (derived from experimental measurements on endothelial cells) 52% euchromatin and 48% heterochromatin distributed with a uniform spatial distribution as described in our previous work [46].

For the irradiations at 40 and 220 kVp, 10,000 electrons taken from the phase space were generated in order to be used as the source, whereas 50,000 electrons were generated for 4 MV in order to always maintain statistical uncertainties (root mean square error (RMSE)) below 6% of the mean number of DSB per track. The electron properties (position in z, momentum, and kinetic energy) were derived from the first stage of the simulation as explained in Section 4.2. Coordinates in the x–y plane were restricted to the cell nucleus dimensions.

##### Computation Chain to Score Strand Breaks

A computation chain [35] using the Geant4-DNA extension of the Geant4 toolkit (version 10.1) was used to perform simulations of physical, physico-chemical, and chemical stages within the DNA structure.

In brief, direct damage to the backbone of the DNA structure (direct simple strand breaks (SSB)) is scored from the addition of the energy deposited by inelastic collisions within the 2-deoxyribose, phosphate, and hydration shell volumes of the same nucleotide. If the cumulated energy is higher than a threshold value of 17.5 eV [67,68,69], a direct SSB is registered at that location of the DNA molecule.

After simulation of the physical interactions (inelastic and elastic collisions) of the electrons in the endothelial cells, simulation of the physico-chemical stage transforms the ionized and excited water molecules surrounding the DNA structure into water radicals, as described in Karamitros et al. [70,71].

In the simulation of the chemical stage, the seven types of water radicals (H^●^, OH^●^, H_2_, H_2_O_2_, OH^−^, H_3_O^+^, and solvated e^−^) are taken into account and interact chemically with each other or with DNA molecules (deoxyribose, phosphate, base, and histone volumes) according to the list of possible reactions given by default in the code as shown in Table 4. In our simulation all these interactions were taken into account and indirect damage to the backbone was scored when an OH^●^ radical interacted with a deoxyribose molecule of the target in 40% of the cases [72,73]. Although reactions between some radicals and the DNA bases were also included in these reactions, these were not detailed enough for a correct description of the base damage, the goal being here to achieve a better evaluation of the available radical concentrations for strand breaks production.

As can be seen from Table 4, the simulation considers anoxic conditions since no reaction involving oxygen is considered. This is an unrealistic assumption that should be corrected in further developments of the computation chain that are currently ongoing. Another important feature of the chemical stage simulation is the way scavengers in the cell medium are taken into account. Indeed, the scavenging capacity of histone is simulated by introducing a specific reaction by which all types of radicals arriving to the sphere representing the histone proteins are removed from the simulation. The second important factor is the duration of the chemical stage that prevents radicals far from the DNA structure from being at the origin of indirect effects. This parameter was set by default to 2.5 ns. However, in our recent work [46] introducing the nucleus model filled with heterochromatin and euchromatin, it was suggested that an end-time of 10 ns would be better adapted in order to reproduce the mean distance travelled by OH^●^ radicals in a cell medium after irradiation. Hence, simulations with a chemistry stage simulation end-time of 10 ns were also performed in this work.

##### Definition of a DSB and DSB Complexity

In this work, a focus was placed on the calculation of simple and double-strand breaks of the DNA backbone and their complexity, which can be more easily correlated to the experimental measurements of foci used for comparison. A double-strand break was defined as at least two strand breaks separated by less than 10 base pairs (bp) and with one of them in an opposite strand to the other(s). Concerning definitions of DSB complexity, different authors have used different definitions [48,74]. As in previous work by our group, the DBScan [75] clustering algorithm was used in order to look at the relative position of all the strand breaks produced in the simulation either in the physical or chemical stage and to calculate the DSBs. Using this algorithm, any other SB separated by less than 10 bp from the initial DSB was merged to create a complex DSB. The DSB complexity was thus given by the number of SBs inside a complex DSB. Our algorithm is therefore not intended to provide non-DSB clusters including other DNA lesions such as base damage.

## 5. Conclusions

In this work, microdosimetric calculations illustrated the distributions of specific energy in an irradiated cell population, and, thus, that the microdosimetric spread $\sigma_{z}^{\mathrm{rel}}$ was similar when considering 40 and 220 kVp but different when looking at 4 MV. With regard to the nanodosimetric aspect, DSB induction in the form of DSB/Gy/Gbp was very similar among 40 kVp and 220 kVp X-rays and higher in comparison with 4 MV by a factor of 1.3, as could be expected from their calculated secondary electron spectra. Nevertheless, DSB complexity was relatively similar. The simulation results presented in this study confirmed results observed in previous experiments led by IRSN radiobiologists, who found no significant differences between 40 kVp and 220 kVp but a higher number of γ-H2AX foci from these radiation qualities compared to 4 MV X-rays for a dose of 2 Gy. From the simulation results, these observations could be explained by the differences in the proportion of low-energy electrons (≤10 keV) between the three X-rays energies.

## Figures and Tables

**Table 1:** Proportion of photoelectric effect (PE), Compton effect (CE), and pair production (CONV) as well as the mean energy of secondary electrons (in keV), for each photon energy spectrum.

Radiation Quality	40 kVp X-rays	220 kVp X-rays	4 MV X-rays
% PE	68.9	19.6	3.3
% CE	31.1	80.4	96.3
% CONV	0	0	0.4
Mean energy of secondary electrons (keV)	9.8	18.4	858.4

**Table 2:** Simulated results in this work: mean number of double-strand breaks (DSB)/Gy/giga base pairs (Gbp) and proportion of DSB complexity for 40, 220 kVp, and 4 MV X-rays. Simple DSBs only contain two strand breaks while complex DSBs contain three or more strand breaks with at least one of them in an opposite strand to the others. Errors from simulations correspond to the standard deviation of the mean over 10 batches.

Radiation Quality	40 kVp X-rays		220 kVp X-rays		4 MV X-rays	
Duration of the chemical stage	2.5 ns	10 ns	2.5 ns	10 ns	2.5 ns	10 ns
DSB/Gy/Gbp	3.5 ± 0.3	4.7 ± 0.3	3.5 ± 0.3	4.7 ± 0.2	2.8 ± 0.3	3.6 ± 0.3
Simple DSB (%)	86.0 ± 3.3	86.3 ± 1.8	86.4 ± 2.1	86.2 ± 2.1	87.7 ± 2.9	86.5 ± 2.3
Complex DSB (%)	14.0 ± 3.3	13.7 ± 1.8	13.6 ± 2.1	13.8 ± 2.1	12.3 ± 2.9	13.5 ± 2.3

**Table 3:** Simulated results and experimental data obtained at IRSN [30]: mean number of γ-H2AX foci per endothelial cell nucleus in Gap0/Gap1 (30 min post-irradiation) and mean number of simulated DSB per nucleus for chemical simulation end-times of 2.5 and 10 ns for a dose of 1 Gy related to the experimental data at 1 Gy for 40 kVp, 220 kVp, and 4 MV X-rays. Errors from simulations correspond to the standard deviation over 10 batches while experimental ones correspond to the standard error.

**Experimental Data**	**40 kVp X-rays**	**220 kVp X-rays**	**4 MV X-rays**
Mean number of γ-H2AX foci per nucleus (30 min post-irradiation) [30]	0.25 Gy: 5.35 ± 1.13	0.25 Gy: 7.35 ± 2.17	0.25 Gy: 4.35 ± 0.21
	0.5 Gy: 9.88 ± 0.87	0.5 Gy: 10.24 ± 1.73	0.5 Gy: 8.54 ± 1.42
	1 Gy: 18.59 ± 0.43	1 Gy: 18.64 ± 2.33	1 Gy: 16.46 ± 1.63
	2 Gy: 30.30 ± 2.21	2 Gy: 30.59 ± 2.96	2 Gy: 26.42 ± 0.87
**Simulated DSBs and Experimental Foci at 1 Gy**	**40 kVp X-rays**	**220 kVp X-rays**	**4 MV X-rays**
Mean number of DSBs per nucleus (sim.) for a chemical simulation end-time of 2.5 ns	21.0 ± 0.3	21.0 ± 0.3	16.8 ± 0.3
Mean number of DSBs per nucleus (sim.) for a chemical simulation end-time of 10 ns	28.2 ± 0.3	28.2 ± 0.2	21.6 ± 0.3
Mean number of γ-H2AX foci per nucleus (exp.)	18.59 ± 0.43	18.64 ± 2.33	16.46 ± 1.63

**Table 4:** Reactions and reaction rates used in the simulation [46]. (**a**) default reactions present in the Geant4-DNA chemistry module and (**b**) reactions added in the simulation.

**(a)**	
**Reaction**	**Reaction Rate (10^10^·M^−1^s^−1^)**
*H^●^ + e^−^_aq_ + H_2_O → OH^−^ + H_2_*	2.65
*H^●^ + OH^●^ → H_2_O*	1.44
*H^●^ + H^●^ → H_2_*	1.20
*H_2_ + OH^●^ → H^●^ + H_2_O*	4.17 × 10^−3^
*H_2_O_2_ + e^−^_aq_ → OH^−^ + OH^●^*	1.41
*H_3_O^+^ + e^−^_aq_ → H^●^ + H_2_O*	2.11
*H_3_O^+^ + OH^−^ → 2H_2_O*	14.3
*OH^●^ + e^−^_aq_ → OH^●^*	2.95
*OH^●^ + OH^●^ → H_2_O_2_*	0.44
*e^−^_aq_ + e^−^_aq_ + 2H_2_O → 2OH^−^ + H_2_*	0.50
**(b)**	
**Reaction**	**Reaction Rate (10^9^·M^−1^s^−1^)**
*2-deoxyribose + OH^●^*	1.8
*Adenine + OH^●^*	6.1
*Guanine + OH^●^*	9.2
*Thymine + OH^●^*	6.4
*Cytosine + OH^●^*	6.1
*2-deoxyribose + e^−^_aq_*	0.01
*Adenine + e^−^_aq_*	9.0
*Guanine + e^−^_aq_*	14.0
*Thymine + e^−^_aq_*	18.0
*Cytosine + e^−^_aq_*	13.0
*2-deoxyribose + H^●^*	0.029
*Adenine + H^●^*	0.10
*Thymine + H^●^*	0.57
*Cytosine + H^●^*	0.092
